# Facility and Regional Factors Associated With the New Adoption of Electronic Medical Records in Japan: Nationwide Longitudinal Observational Study

**DOI:** 10.2196/14026

**Published:** 2019-06-14

**Authors:** Hideaki Kawaguchi, Soichi Koike, Kazuhiko Ohe

**Affiliations:** 1 Department of Biomedical Informatics The University of Tokyo Tokyo Japan; 2 Division of Health Policy and Management Center for Community Medicine Jichi Medical University Tochigi Japan

**Keywords:** electronic health records, health services research, health policy, Bayes theorem

## Abstract

**Background:**

The rate of adoption of electronic medical record (EMR) systems has increased internationally, and new EMR adoption is currently a major topic in Japan. However, no study has performed a detailed analysis of longitudinal data to evaluate the changes in the EMR adoption status over time.

**Objective:**

This study aimed to evaluate the changes in the EMR adoption status over time in hospitals and clinics in Japan and to examine the facility and regional factors associated with these changes.

**Methods:**

Secondary longitudinal data were created by matching data in fiscal year (FY) 2011 and FY 2014 using reference numbers. EMR adoption status was defined as “EMR adoption,” “specified adoption schedule,” or “no adoption schedule.” Data were obtained for hospitals (n=4410) and clinics (n=67,329) that had no adoption schedule in FY 2011 and for hospitals (n=1068) and clinics (n=3132) with a specified adoption schedule in FY 2011. The EMR adoption statuses of medical institutions in FY 2014 were also examined. A multinomial logistic model was used to investigate the associations between EMR adoption status in FY 2014 and facility and regional factors in FY 2011. Considering the regional variations of these models, multilevel analyses with second levels were conducted. These models were constructed separately for hospitals and clinics, resulting in four multinomial logistic models. The odds ratio (OR) and 95% Bayesian credible interval (CI) were estimated for each variable.

**Results:**

A total of 6.9% of hospitals and 14.82% of clinics with no EMR adoption schedules in FY 2011 had adopted EMR by FY 2014, while 10.49% of hospitals and 33.65% of clinics with specified adoption schedules in FY 2011 had cancelled the scheduled adoption by FY 2014. For hospitals with no adoption schedules in FY 2011, EMR adoption/scheduled adoption was associated with practice size characteristics, such as number of outpatients (from quantile 4 to quantile 1: OR 1.67, 95% CI 1.005-2.84 and OR 2.40, 95% CI 1.80-3.21, respectively), and number of doctors (from quantile 4 to quantile 1: OR 4.20, 95% CI 2.39-7.31 and OR 2.02, 95% CI 1.52-2.64, respectively). For clinics with specified EMR adoption schedules in FY 2011, the factors negatively associated with EMR adoption/cancellation of scheduled EMR adoption were the presence of beds (quantile 4 to quantile 1: OR 0.57, 95% CI 0.45-0.72 and OR 0.74, 95% CI 0.58-0.96, respectively) and having a private establisher (quantile 4 to quantile 1: OR 0.27, 95% CI 0.13-0.55 and OR 0.43, 95% CI 0.19-0.91, respectively). No regional factors were significantly associated with the EMR adoption status of hospitals with no EMR adoption schedules; population density was positively associated with EMR adoption in clinics with no EMR adoption schedule (quantile 4 to quantile 1: OR 1.49, 95% CI 1.32-1.69).

**Conclusions:**

Different approaches are needed to promote new adoption of EMR systems in hospitals as compared to clinics. It is important to induce decision making in small- and medium-sized hospitals, and regional postdecision technical support is important to avoid cancellation of scheduled EMR adoption in clinics.

## Introduction

With the increasing focus on electronic health, the secondary utilization of electronic medical records (EMRs) is becoming important as a tool for collecting patient clinical information. Data mining methods for generating new knowledge from large datasets and machine learning methods (such as deep learning) are developing rapidly, and the use of medical information has accordingly attracted more attention [1].

Although the rate of adoption of the EMR system has increased internationally, the adoption rate in Japan is lower than that in other countries [2-5]. Furthermore, the Survey of Medical Institutions conducted by the Ministry of Health, Labour and Welfare in fiscal year (FY) 2014 revealed that 45.5% of hospitals and 60.8% of clinics are not planning to adopt EMRs in the future [6], indicating that the lack of new adoption of EMRs is a major issue in Japan.

Resistance to new adoption of EMRs is not specific to Japan. For example, while financial incentives such as the Meaningful Use program have contributed to the growing EMR adoption rate in the United States [7], the growth rate is slowing down [8] and the adoption rates in underserved and small hospitals are lower than those in other hospitals [9,10]. To promote further EMR adoption, it is necessary to analyze the characteristics of medical facilities that have newly adopted the EMR system.

Although previous studies have revealed the characteristics of medical institutions that have adopted EMR systems [11-15], to the best of our knowledge, no study has used time series data to analyze the factors directly related to new adoption of EMRs. To predict whether medical institutions will adopt the EMR system in the near future, it is necessary to analyze longitudinal data rather than cross-sectional data. Furthermore, the EMR adoption process consists of several stages, and therefore, decision making for EMR adoption should be considered separately from management after the decision to adopt EMRs has been made [16]. Therefore, research should evaluate the changes over time in the EMR adoption status and determine the factors associated with these changes. In addition to facility factors, geographical or regional factors affecting the new adoption of EMR should be considered. Previous studies have reported regional variation in EMR adoption and associations between EMR adoption and health care professional shortage area and metropolitan statuses [17,18]. Furthermore, the regional factors associated with EMR adoption differ between hospitals and clinics in Japan [19]. Thus, we hypothesized that the changes in EMR adoption status over time and the factors associated with these changes would differ between hospitals and clinics. To test this hypothesis, this study aimed to evaluate the changes in the EMR adoption status of hospitals and clinics in Japan over time and to evaluate the facility and regional factors associated with these changes.

## Methods

### Study Design

This study was a nationwide longitudinal observational study that secondarily analyzed existing survey data. Data for FY 2011 and FY 2014 were matched using reference numbers, creating longitudinal data from the whole of Japan.

**Figure 1:**
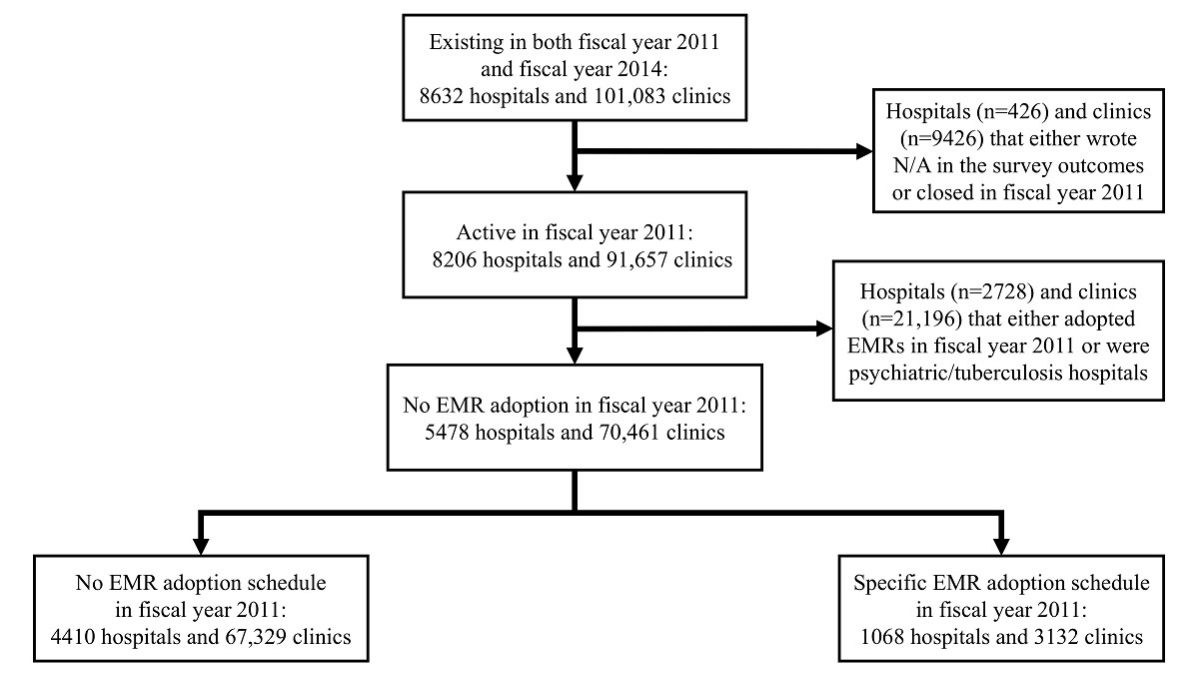
Inclusion/exclusion criteria of hospitals and clinics. EMR: electronic medical record; N/A: not applicable.

As in a previous study [16], the two steps involved in EMR adoption were assumed to be (1) having no adoption schedule to deciding to adopt EMRs and (2) deciding to adopt an EMR system for successful EMR adoption. The EMR adoption status in FY 2014 was examined in medical institutions that had no adoption schedule in FY 2011 and in medical institutions that had scheduled EMR adoption in FY 2011. This investigation included all the hospitals and clinics in Japan that had not adopted EMRs in FY 2011. A “clinic” in Japan was defined as a medical institution with fewer than 20 beds. First, these hospitals and clinics were divided into those that had not scheduled EMR adoption in FY 2011 and those that had scheduled EMR adoption in FY 2011. Second, the medical institutions were assessed in accordance with the inclusion/exclusion criteria in Figure 1. This study finally analyzed 4410 hospitals and 67,329 clinics that had not scheduled EMR adoption in FY 2011 and 1068 hospitals and 3132 clinics that had scheduled EMR adoption in FY 2011.

### Data Sources

Data on EMR adoption and other characteristics of medical facilities were obtained from the Survey of Medical Institutions, which is a detailed triennial survey of all medical institutions conducted by the Japan Ministry of Health, Labour and Welfare [6]. Permission was obtained from the Japan Ministry of Health, Labour and Welfare to analyze the survey data of individual medical institutions. The EMR adoption status of each medical facility was defined as a response of 1/2, 3, or 4 to the survey item “Electronic medical record system adoption status,” where 1=adopted in the entire hospital/clinic, 2=adopted in part of the hospital/clinic, 3=specified adoption schedule, and 4=no adoption schedule.

Geographical or regional information, such as municipality boundary data, was obtained from the Municipality Map Maker for Web [20]. Japan comprises 47 prefectures, and the Japanese Government established subprefectural medical regions called secondary medical service areas (SMSAs) [19]. An SMSA is defined as a medical unit that evaluates the demand and supply of health resources. To determine the SMSA data, ArcGIS version 10.2.1 (ESRI Japan Inc, Tokyo, Japan) was used to combine municipality-level parameters, as each SMSA consists of several municipalities. The assessed regional factors included available socioeconomic and macro health-environment factors identified in previous studies [17-19]; these data were collected from e-Stat, the national Japanese government database [21].

### Statistical Analysis

#### Multinomial Logistic Regression Analysis

A multinomial logistic model was used to investigate the associations between EMR adoption status in FY 2014 and facility and regional factors in FY 2011. Multinomial logistic regression analysis is a statistical model that deals with more than three categorical variables. Three EMR adoption statuses (ie, EMR adoption, specified adoption schedule, and no adoption schedule) were set as outcome variables. It is also necessary to determine a reference in advance regarding outcome variables in the multinomial logistic regression model. “No adoption schedule” was set in advance as a reference regarding outcome variables in the model targeting medical institutions that had not scheduled EMR adoption in FY 2011 (model 1), and “specified adoption schedule” was set as a reference in the model targeting medical institutions that had scheduled EMR adoption in FY 2011 (model 2). These models were constructed separately for hospitals and clinics, resulting in four multinomial logistic models.

#### Multilevel Analysis

To take regional variations of these models into account, multilevel analyses with second levels were conducted. Random variations in intercepts at the SMSA level were set as the second level. Four multilevel multinomial logistic regression models were constructed.

### Explanatory Variables

The facility variables used in this study comprised facility factors identified in previous studies [11-15], which were collected from the Survey of Medical Institutions in FY 2011. These factors were advocating internal medicine, advocating surgery, emergency medical institution design, number of outpatients, number of doctors, presence of interns, implementation of home medical care, classification of the establisher, and number of beds. The presence of interns was not included as an explanatory variable in the models targeting clinics, as clinics rarely have interns in Japan. As the numbers of outpatients were extremely skewed, medical institutions were categorized in accordance with the number of outpatients from quantile 1 (lowest) to quantile 4 (highest). Regarding the classification of the establisher, “national,” “public medical institution,” and “social insurance affiliated organization” were defined as public, while “medical corporation,” “private,” and “others” were defined as private. Hospitals were categorized in accordance with the number of doctors from quantile 1 (lowest) to quantile 4 (highest), while clinics were categorized into those with more than one doctor or those with less than one doctor. In accordance with the format of the Survey of Medical Institutions, the number of doctors in hospitals refers to the number of working doctors, while the number of doctors in clinics refers to those employed on a full-time basis. Hospitals were categorized into those with less than 200 beds, 200-399 beds, and ≥400 beds, while clinics were categorized into those with or without beds.

The following regional factors were analyzed: population density (people per km^2^), average per capita income (million JPY), number of working doctors per 1000 people (separately for hospitals and clinics), and proportion of interns to all working doctors. As the population density distribution was extremely skewed, medical institutions were categorized in accordance with the population density from quantile 1 (lowest density) to quantile 4 (highest density). As the two surveys were conducted in different years, the data for population density and average per capita income were obtained for FY 2010. Data with missing values in explanatory variables were deleted.

### Parameter Estimations

Markov chain Monte Carlo simulations with 1000 iterations and a burn-in period of 500 iterations were used to estimate the parameters of the multilevel multinomial logistic models. R-hat diagnostic was used to check Markov chain Monte Carlo convergence, with 1.1 set as the cut-off value [22]. The odds ratio (OR) and 95% Bayesian credible intervals (CI) were calculated for each variable, and an association was considered nonsignificant if the 95% CI of the OR included 1.

The multicollinearity of covariates was evaluated using the generalized variance inflation factor (GVIF) [23]. Although the average per capita income was an important factor, this factor was removed because it had the greatest GVIF of >2.5. Furthermore, the number of doctors was removed from the model targeting hospitals with a specified EMR adoption schedule because this factor had a high GVIF of >2.5. All other variables had a GVIF of <2.5 and were entered into the multilevel multinomial logistic model.

The widely applicable information criterion (WAIC) was used as a measure of the goodness-of-fit of the Bayesian statistical model; the model with the lowest WAIC was considered the best-fit model [24]. The WAIC was used to compare the multilevel multinomial logistic regression model with a normal multinomial logistic regression model without consideration of the SMSA-level effects.

All analyses were conducted using R V.3.4.1 (R Core Team, Vienna, Austria) [25].

## Results

### Time Series Changes in the Electronic Medical Records Adoption Status of Hospitals and Clinics

Table 1 shows the status of EMR adoption in FY 2014. Only 6.9% of the hospitals with no EMR adoption schedule in FY 2011 had newly adopted EMRs by FY 2014, while 14.82% of clinics with no EMR adoption schedule in FY 2011 had newly adopted EMRs by FY 2014. However, 10.49% of hospitals with a specified adoption schedule in FY 2011 had cancelled the scheduled adoption by FY 2014, while 33.65% of clinics with a specified adoption schedule in FY 2011 had cancelled the scheduled adoption by FY 2014.

### Multilevel Multinomial Logistic Regression Targeting Hospitals

Table 2 shows the associations between the EMR adoption status and each explanatory variable for hospitals. After removing the hospitals with missing data, the model included 4278 hospitals with no adoption schedule and 1051 hospitals with a specified adoption schedule in FY 2011.

For hospitals with no adoption schedule in FY 2011, the factors associated with EMR adoption and specified adoption schedules were the number of doctors, number of outpatients, and presence of interns. The number of outpatients was more strongly associated with EMR adoption, while the number of doctors and presence of interns were more strongly associated with specified adoption schedules.

For hospitals with specified adoption schedules in FY 2011, the number of outpatients, number of beds, presence of interns, and population density were associated with EMR adoption, while advocating surgery was associated with the cancellation of scheduled EMR adoption.

The WAICs of the multilevel models with consideration of regional effects targeting hospitals with no adoption schedule in FY 2011 and hospitals with specified adoption schedules in FY 2011 were 6538.6 and 1859.7, respectively; those of the regression models without consideration of regional effects were 6548.4 and 1859.9, respectively. This indicates that the multilevel models did not produce a much better fit than the normal regression models without consideration of the regional effects.

**Table 1:** Electronic medical record adoption status in fiscal year 2014.

Facility	No adoption schedule in fiscal year 2011			Specified adoption schedule in fiscal year 2011		
	Adoption, n (%)	Specified adoption schedule, n (%)	No adoption schedule, n (%)	Adoption, n (%)	Specified adoption schedule, n (%)	No adoption schedule, n (%)
Hospitals	303 (6.87)	1212 (27.48)	2895 (65.65)	563 (52.72)	393 (36.80)	112 (10.49)
Clinics	9981 (14.82)	3045 (4.52)	54303 (80.65)	1360 (43.42)	718 (22.92)	1054 (33.65)

**Table 2:** Results of multilevel multinomial logistic regression targeting hospitals. Significant variables are presented as italics.

Target		Hospitals with no adoption schedule in fiscal year 2011 (n=4278)		Hospitals with a specified adoption schedule in fiscal year 2011 (n=1051)	
		Adoption, OR^a^ (95% CI^b^)	Specified adoption schedule, OR (95% CI)	Adoption, OR (95% CI)	No adoption schedule, OR (95% CI)
Intercept		0.06 (0.03-0.14)	0.19 (0.13-0.31)	0.96 (0.42-2.19)	0.57 (0.16-1.86)
Advocating internal medicine		0.76 (0.46-1.29)	0.85 (0.63-1.12)	0.94 (0.51-1.78)	1.41 (0.60-3.83)
Advocating surgery		1.11 (0.76-1.58)	0.97 (0.81-1.17)	1.03 (0.68-1.58)	*0.51 (0.28*-*0.90)*
Designed as an emergency hospital		1.38 (0.999-1.94)	1.20 (0.99-1.45)	1.06 (0.74-1.56)	1.36 (0.77-2.47)
**Number of outpatients**					
	Quantile 1	1.00 (reference)	1.00 (reference)	1.00 (reference)	1.00 (reference)
	Quantile 2	0.77 (0.47-1.23)	1.18 (0.94-1.49)	*1.83 (1.21*-*2.83)*	0.98 (0.53-1.72)
	Quantile 3	1.04 (0.63-1.66)	*1.68 (1.31-2.14)*	*2.84 (1.82-4.40)*	*0.43 (0.20-0.88)*
	Quantile 4	*1.67 (1.005-2.84)*	*2.40 (1.80-3.21)*	*2.79 (1.60-5.14)*	0.58 (0.21-1.44)
**Number of doctors^c^**					
	Quantile 1	1.00 (reference)	1.00 (reference)	N/A^d^	N/A
	Quantile 2	1.58 (0.99-2.55)	1.13 (0.90-1.40)	N/A	N/A
	Quantile 3	*1.86 (1.13-3.08)*	*1.54 (1.23-1.95)*	N/A	N/A
	Quantile 4	*4.20 (2.39-7.31)*	*2.02 (1.52-2.64)*	N/A	N/A
Presence of interns		*2.08 (1.34-3.16)*	*1.45 (1.07-1.95)*	*1.87 (1.28-2.86)*	0.88 (0.38-2.02)
Implementation of home medical care		0.96 (0.72-1.25)	*1.18 (1.01-1.39)*	0.93 (0.69-1.26)	0.70 (0.44-1.13)
Private establisher		0.73 (0.51-1.08)	0.85 (0.67-1.07)	0.73 (0.50-1.08)	0.74 (0.37-1.50)
**Number of beds**					
	<200	1.00 (reference)	1.00 (reference)	1.00 (reference)	1.00 (reference)
	200-399	1.19 (0.82-1.71)	1.12 (0.88-1.42)	1.18 (0.80-1.76)	0.94 (0.48-1.75)
	≥400	1.38 (0.72-2.58)	1.34 (0.83-2.17)	*2.10 (1.12-3.86)*	0.81 (0.24-2.37)
**Population density per km^2^**					
	Quantile 1	1.00 (reference)	1.00 (reference)	1.00 (reference)	1.00 (reference)
	Quantile 2	0.84 (0.56-1.23)	1.13 (0.91-1.41)	0.78 (0.49-1.25)	0.71 (0.35-1.56)
	Quantile 3	1.02 (0.68-1.57)	1.25 (0.98-1.58)	0.75 (0.47-1.21)	0.79 (0.37-1.80)
	Quantile 4	0.80 (0.50-1.27)	0.97 (0.76-1.25)	*0.44 (0.25-0.78)*	1.09 (0.49-2.54)
Working doctors per 1000 population		1.08 (0.87-1.35)	1.07 (0.94-1.21)	1.05 (0.87-1.28)	0.84 (0.56-1.18)
Proportion of interns to all working doctors		0.94 (0.88-1.002)	0.98 (0.95-1.02)	0.99 (0.92-1.07)	1.03 (0.90-1.16)

^a^OR: odds ratio.

^b^CI: credible interval.

^c^The factor “number of doctors” was removed from the model targeting hospitals with a specified electronic medical record adoption schedule, as it had a high generalized variance inflation factor of >2.5

^d^N/A: not applicable.

### Multilevel Multinomial Logistic Regression Targeting Clinics

Table 3 shows the associations between EMR adoption status and each explanatory variable for clinics. After removing the clinics with missing data, the model included 55,815 clinics with no adoption schedule and 3030 clinics with a specified adoption schedule in FY 2011.

**Table 3:** Results of multilevel multinomial logistic regression targeting clinics. Significant variables are presented as italics.

Target		Clinics with no adoption schedule in fiscal year 2011 (n=55,815)		Clinics with a specified adoption schedule in fiscal year 2011 (n=3030)	
		Adoption, OR^a^ (95% CI^b^)	Specified adoption schedule, OR (95% CI)	Adoption, OR (95% CI)	No adoption schedule, OR (95% CI)
Intercept		0.07 (0.06-0.08)	0.02 (0.01-0.02)	7.05 (3.34-15.19)	6.87 (3.21-15.46)
Advocating internal medicine		*1.17 (1.11-1.23)*	*1.26 (1.15-1.38)*	0.99 (0.79-1.22)	0.84 (0.66-1.05)
Advocating surgery		1.06 (0.99-1.14)	1.10 (0.998-1.22)	1.07 (0.84-1.35)	0.98 (0.76-1.25)
Designed as an emergency clinic		0.85 (0.55-1.29)	1.14 (0.71-1.81)	0.72 (0.26-2.01)	1.23 (0.46-3.34)
**Number of outpatients**					
	Quantile 1	1.00 (reference)	1.00 (reference)	1.00 (reference)	1.00 (reference)
	Quantile 2	*1.26 (1.17-1.35)*	*1.67 (1.47-1.90)*	0.94 (0.72-1.25)	*0.68 (0.52-0.91)*
	Quantile 3	*1.33 (1.24-1.44)*	*2.05 (1.81-2.33)*	0.88 (0.67-1.16)	*0.51 (0.39-0.68)*
	Quantile 4	*1.52 (1.41-1.64)*	*2.61 (2.29-2.95)*	0.86 (0.65-1.14)	*0.47 (0.34-0.63)*
More than one doctor		*1.30 (1.24-1.37)*	*1.94 (1.79-2.10)*	1.21 (0.98-1.48)	0.88 (0.71-1.08)
Implementation of home medical care		*1.15 (1.09-1.21)*	*1.48 (1.35-1.62)*	0.93 (0.75-1.15)	0.87 (0.70-1.08)
Private establisher		*1.18 (1.04-1.35)*	0.90 (0.75-1.11)	*0.27 (0.13-0.55)*	*0.43 (0.19-0.91)*
Presence of beds		*0.89 (0.82-0.97)*	*1.20 (1.08-1.33)*	*0.57 (0.45-0.72)*	*0.74 (0.58-0.96)*
**Population density per km^2^**					
	Quantile 1	1.00 (reference)	1.00 (reference)	1.00 (reference)	1.00 (reference)
	Quantile 2	*1.14 (1.04-1.26)*	1.07 (0.94-1.19)	0.92 (0.71-1.21)	0.84 (0.63-1.11)
	Quantile 3	*1.35 (1.22-1.50)*	1.05 (0.92-1.20)	1.07 (0.79-1.45)	1.18 (0.89-1.60)
	Quantile 4	*1.49 (1.32-1.69)*	1.14 (0.99-1.30)	1.11 (0.79-1.54)	1.26 (0.92-1.76)
Working doctors per 1000 population		0.98 (0.93-1.03)	1.03 (0.98-1.08)	0.92 (0.83-1.03)	0.93 (0.84-1.02)
Proportion of interns to all working doctors		1.01 (0.995-1.03)	1.00 (0.98-1.02)	1.04 (0.99-1.09)	1.02 (0.97-1.07)

^a^OR: odds ratio.

^b^CI: credible interval.

For clinics with no adoption schedule in FY 2011, a wider range of factors (such as private establisher and population density) were associated with EMR adoption than with specified adoption schedules.

For clinics with specified adoption schedules in FY 2011, the presence of beds and having a private establisher were associated with both EMR adoption and the cancellation of scheduled EMR adoption. In contrast, the number of outpatients was negatively associated with the cancellation of scheduled EMR adoption.

The WAICs of the multilevel models with consideration of regional effects targeting clinics with no adoption schedule in FY 2011 and clinics with specified adoption schedules in FY 2011 were 65477.2 and 6411.3, respectively; those of the regression models without consideration of regional effects were 65615.6 and 6416.3, respectively. This indicates that the multilevel model targeting clinics with no adoption schedule in FY 2011 had a slightly better fit than the normal regression model without consideration of regional effects.

## Discussion

### Time Series Changes in the Electronic Medical Records Adoption Status in Hospitals Versus Clinics

Time series data were used to precisely analyze the changes over time in the EMR adoption status and the factors affecting new EMR adoption in hospitals and clinics in Japan. To the best of our knowledge, this is the first study to detail the changes in the EMR adoption status over time.

Fewer hospitals with no EMR adoption schedule in FY 2011 had adopted EMR within 3 years compared with clinics with no EMR adoption schedules in FY 2011. However, more hospitals with no EMR adoption schedule in FY 2011 had planned to adopt EMR within 3 years compared with clinics without an EMR adoption schedule in FY 2011. This shows that more hospitals than clinics planned to adopt EMR, but that clinics took less time to adopt EMR than hospitals. As 37,876 or more clinics were staffed by a maximum of one physician, such clinics would be able to implement EMR more quickly than hospitals, and this difference in implementation speed may have influenced the increased incidence of new EMR adoption in clinics compared with hospitals. Furthermore, only few clinics planned to but did not adopt EMR within 3 years (4.52%). Although such clinics can make decisions relatively easily, it is likely that their EMR adoption capabilities are lacking; thus, follow-up on the implementation of EMR is necessary.

About half of the hospitals with specified EMR adoption schedules in FY 2011 had actually adopted EMR within 3 years, while about 10% had cancelled the scheduled EMR adoption within 3 years. In contrast, a greater proportion of clinics with specified EMR adoption schedules in FY 2011 had cancelled the scheduled EMR adoption within 3 years (33.65%). The reason why more clinics than hospitals cancelled the scheduled EMR adoption might be that clinics are also quicker to make decisions to cancel scheduled adoption than hospitals.

In summary, once the decision to adopt an EMR system has been made, hospitals tend to follow through with scheduled EMR adoption more often than clinics; therefore, the decision-making process itself seems to be important for hospitals. However, compared with hospitals, more clinics decided to adopt EMR and then cancelled the scheduled EMR adoption; therefore, adequate EMR adoption support after decision making seems to be important for clinics.

### Facility Factors Associated With the Electronic Medical Records Adoption Status of Hospitals Versus Clinics

For hospitals with no EMR adoption scheduled in FY 2011, the facility factors associated with actual EMR adoption were also associated with the scheduling of EMR adoption. In particular, the EMR system was adopted more often by hospitals with large numbers of medical staff, which is consistent with previous studies [11,26,27]. In addition, multilevel multinomial logistic regression targeting hospitals with specified EMR adoption schedules in FY 2011 revealed that medium-sized hospitals rarely cancelled scheduled EMR adoption. Therefore, it is important to encourage small- or medium-sized hospitals to adopt the EMR system. For example, the implementation of financial incentives such as the Meaningful Use program might effectively increase the decision to adopt the EMR system, as in the United States [7,28]. In Japan, financial incentives for EMR adoption have been offered to large hospitals [5], but these also need to be offered to small- and medium-sized hospitals.

Regarding clinics with no EMR adoption schedule in FY 2011, large clinics with a large number of outpatients and more than one doctor, similar to hospitals, were more likely to adopt and plan to adopt the EMR system compared with small clinics. Furthermore, the implementation of home medical care was a significant factor influencing EMR adoption, and the use of EMRs might be expected to prompt sharing of medical information, as in the United States [8,29]. Of the clinics with no EMR adoption schedule in FY 2011, those that had beds were more likely to plan to adopt EMR, while those without beds were more likely to actually adopt EMR. In addition, multilevel multinomial logistic regression targeting clinics with a specified EMR adoption schedule in FY 2011 revealed that the clinics that had beds tended not to adopt the EMR system and not to cancel the EMR adoption schedule. In other words, clinics that had beds tended to postpone the scheduled EMR adoption, despite being more likely to adopt the EMR system. Therefore, postdecision support might be particularly useful for clinics with beds.

### Regional Factors Associated With the Electronic Medical Records Adoption Status of Hospitals Versus Clinics

Regional factors were not associated with the EMR adoption status of hospitals with no EMR adoption schedule in FY 2011. In addition, the WAIC of the model that considered the SMSA-level effects was close to that of the model that did not consider SMSA-level effects, indicating that regionality did not have a large influence on the EMR adoption status of hospitals. This is consistent with our previous study [19]. For hospitals, the new adoption of EMR was mainly influenced by facility factors rather than regional factors.

Regarding clinics, population density was positively associated with EMR adoption in clinics with no EMR adoption schedule in FY 2011, and the WAIC of the model that considered the SMSA-level effects was less than that of the model that did not consider SMSA-level effects, indicating that regionality influences EMR adoption in clinics. These results are also consistent with our previous research [19]. An example of EMR adoption support on a regional basis is the Regional Extension Centers program in the United States [18,30], which provides technical support for EMR implementation, mainly in rural areas. Development of the Regional Extension Centers program might lead to the expansion of regional health care networks in Japan.

### Trends in Electronic Medical Records Adoption After 2015

Although this study used the most recent available data (from FY 2014), the status of EMR usage is progressing rapidly. Therefore, the trends regarding EMR adoption after 2015 are described here and compared with the results of this study.

In the United States, the EMR adoption rate has rapidly grown, and the Health Information Technology for Economic and Clinical Health act has received a certain appreciation [7]. Similarly, EMR adoption has advanced via the distribution of financial incentives in other countries. For example, financial incentives are considered important for the adoption of national EMR in France [31] and Canada [32]. This suggests that our findings are consistent with the EMR adoption trends in other countries after 2015.

Although it was not possible to include the data of individual medical institutions from the Survey of Medical Institutions conducted in FY 2017 in Japan, in this study, we compared the aggregated public data from FY 2017 with the aggregated data from FY 2011 and FY 2014 [6] (Multimedia Appendix 1). Although the changes from FY 2011 to 2014 and from FY 2014 to 2017 regarding clinics showed similar trends, the trends regarding hospitals differed between time periods. Specifically, the decrease in the proportion of hospitals with no EMR adoption schedule in FY 2014-2017 was smaller than that in FY 2011-2014, and the proportion of hospitals with no EMR adoption schedule in FY 2017 was smaller than that in FY 2014. Although a certain number of hospitals with specific EMR adoption schedules in FY 2014 could have implemented EMR adoption by FY 2017, only a small number of hospitals with no EMR adoption schedules in FY 2014 could have adopted EMRs by FY 2017. Therefore, the results of this study regarding hospitals with no EMR adoption schedule in FY 2011 might not be directly applicable to the period from FY 2014 to 2017.

### Limitations

This study has several limitations. First, our study used secondary data, and several data were unavailable. For example, it was not possible to consider important factors such as the characteristics and attitude toward EMR of working physicians, and the profits of the medical institutions. Second, as this study used secondary data sources, the time period was set as 3 years, and the change in EMR adoption status within 3 years was not evaluated. However, using this 3-year period to evaluate the change in EMR adoption status might be too short for hospitals and too long for clinics. In the future, cohort data should be prepared to analyze shorter time periods. Third, although data were acquired from all medical institutions in Japan and generalizability was secured, the number of samples accordingly increased and the regression coefficients tended to be significant; therefore, it might be difficult to interpret the regression coefficients. In particular, there were 10 times more clinics than hospitals. Our results require validation via comparison with other survey data. Fourth, our study used data from up to FY 2014, and therefore, these results cannot explain the EMR adoption situation after FY 2015. Although the recent trends in EMR adoption were described and compared with the results, it is necessary to conduct ongoing research using data from FY 2017.

Despite these limitations, to our knowledge, this is the first study to use longitudinal and spatial data to perform a detailed analysis of the facility and regional factors related to new adoption of EMR in Japan. Although many other countries do not have data available on the EMR adoption status in each hospital [2], this study has the advantage of using time series data obtained from almost all medical institutions in Japan. Our findings will help effectively promote new adoption of the EMR system.

### Conclusions

As the characteristics of time series changes in EMR adoption differ between hospitals and clinics, different approaches are important for the promotion of new adoption of EMRs in hospitals versus clinics in Japan. For hospitals, it is important to induce decision making; for clinics, in addition to inducing decision making, it is important to provide postdecision technical support. In addition, facility factors affecting EMR adoption should mainly be considered for hospitals, while both regional and facility factors should be considered for clinics.

## Appendix

Multimedia Appendix 1 Aggregated data regarding the electronic medical record adoption status in fiscal years 2011, 2014, and 2017. Values are given as n (%).

## Abbreviations

CI: Bayesian credible interval
EMR: electronic medical record
FY: fiscal year
GVIF: generalized variance inflation factor
OR: odds ratio
SMSA: secondary medical service area
WAIC: widely applicable information criterion
