# Supplementary material for: Facility and Regional Factors Associated With the New Adoption of Electronic Medical Records in Japan: Nationwide Longitudinal Observational Study
Source: JMIR Med Inform. 2019 Jun 14;7(2):e14026. doi: 10.2196/14026 (PMC6598416; doi:10.2196/14026)
Supplement: Multimedia Appendix 1 [file medinform_v7i2e14026_app1.pdf]

Multimedia Appendix 1. Aggregated data regarding the electronic medical record adoption status in fiscal years 2011, 2014, and 2017.

	**Hospitals**			**Clinics**		
	**Adoption**	**Specified adoption schedule**	**No adoption schedule**	**Adoption**	**Specified adoption schedule**	**No adoption schedule**
**2011**	1,729 (20.44%)	1,230 (14.54%)	5,501 (65.02%)	20,797 (21.22%)	3,434 (3.50%)	73,773 (75.28%)
**2014**	2,733 (32.18%)	1,897 (22.34%)	3,863 (45.48%)	35,178 (35.02%)	4,244 (4.22%)	61,039 (60.76%)
**2017**	3,693 (43.90%)	1,592 (18.93%)	3,127 (37.17%)	42,167 (41.56%)	4,697 (4.63%)	54,607 (53.82%)

Values are given as n (%).
